# The effectiveness and harms of bortezomib in combination with chemotherapy for mantle cell lymphoma: A protocol for systematic review and meta-analysis

**DOI:** 10.1097/MD.0000000000020961

**Published:** 2020-07-10

**Authors:** Xiaoxia Wang, Wen Li, Xin Wang, Xiaoli Liu, Cuijuan Feng, Yihan Li, Jing Li

**Affiliations:** a Department of Pharmacy, Gansu Gem Flower Hospital, Xigu District; b Department of Oncology, Gansu Gem Flower Hospital, No. 733, Fuli Road, Xigu District; c The Second People's Hospital of Lan Zhou, Chengguan District; d Gansu Health Vocational College, Lanzhou New District; e Emergency Department, Gansu Gem Flower Hospital, No. 733, Fuli Road, Xigu District, Lanzhou City, Gansu Province, China.

**Keywords:** bortezomib, chemotherapy, mantle cell lymphoma, meta-analysis

## Abstract

**Background:**

Chemotherapy with or without consolidation followed by autologous hematopoietic stem cell transplantation is the first-line treatment for mantle cell lymphoma. However, the effectiveness and safety of bortezomib-based chemotherapy for patients with mantle cell lymphoma is still uncertain.

**Methods:**

In this systematic review, the electronic databases of Cochrane Central Register of Controlled Trials, EMBASE, and PUBMED will be searched from inception to May 1, 2020. Randomized controlled trials that assessed the effectiveness and safety of bortezomib in combination with chemotherapy for patients with mantle cell lymphoma will be included. The patient's important outcomes include overall survival, progression-free survival, overall response rate, quality of life, and serious adverse events (eg, grade III-IV peripheral neuropathy, neutropenia, and infection). All process of the study selection, data extraction, and methodology evaluation will be carried out by 2 authors independently. RevMan 5.3 software will be utilized for statistical analysis.

**Results:**

This study will provide a detailed summary of latest evidence related to the effectiveness and safety of bortezomib in combination with chemotherapy in overall survival, progression-free survival, overall response rate, quality of life, and serious adverse events for patients with mantle cell lymphoma

**Conclusion:**

The findings of this study may provide possible guidance for bortezomib in combination with chemotherapy for patients with mantle cell lymphoma.

**Systematic review registration:**

PROSPERO CRD 42020154938.

## 1 Introduction

Mantle-cell lymphoma (MCL) is an incurable, aggressive hematologic cancer with a poor prognosis.^[1,2]^ It comprises 5% to 6% of all non-Hodgkin lymphomas, including approximately 5000 cases per year in the United States.^[3]^

High-dose chemotherapy with or without consolidation followed by autologous hematopoietic stem cell transplantation (ASCT) is the first-line treatment for MCL patient.^[2]^ For patients not suitable for high-dose chemotherapy or transplant, reduced-dose chemotherapy is recommended.^[2,4]^ However, there are no generally accepted therapeutic approaches to date. Combined chemotherapy regimens like cyclophosphamide, doxorubicin, vincristine, prednisone, and rituximab or rituximab, hyperfractionated cyclophosphamide, vincristine, doxorubicin, and dexamethasone, and/or high-dose consolidation therapies, are frequently used.

However, the median failure-free survival for standard therapy is only 8 to 20 months and the median survival of patients with high-intensity chemotherapy is 3 to 4 years.^[5]^ A number of novel agents were later approved for MCL, including bortezomib, lenalidomide, and ibrutinib. Among them, ibrutinib obtained the most significant effects with over 60% overall response rate and almost 20% complete remission in relapsed/refractory MCL,^[6]^ but it is not widely available for patients in developing countries with expensive costs. Lenalidomide did not benefit MCL patients.^[7]^

Bortezomib was confirmed to have a durable response and a favorable rate of progression-free survival (PFS) in single-agent data for relapsed/refractory MCL in a multicenter phase II study,^[8]^ which contributed to it being approved by the FDA for the treatment of MCL patients in relapse after prior therapy. The SWOG trial further showed that the combination of bortezomib with chemotherapy followed by bortezomib maintenance obtained a doubled 2-year PFS rate compared with the chemotherapy regimen alone (62% vs 30%) in previously untreated MCL patients.^[9]^ However, a randomized phase II study assessed the efficacy of bortezomib plus chemotherapy versus chemotherapy in relapsed MCL patients and showed that bortezomib-based chemotherapy had a nonsignificant improvement on PFS (16.5 months vs 8.1 months; *P* = .12).^[10]^

To obtain a better understanding of bortezomib combination therapy in MCL patients, we are planning to perform a meta-analysis of clinical trials to compare the efficacy and safety of bortezomib-based chemotherapy in MCL patients.

## 2 Methods

The guidelines for this systematic review were based on preferred reporting items for systematic reviews and meta-analysis recommendations, and a protocol for this review was published in PROSPERO with the registration number CRD42020154938. Systematic Reviews of Interventions and the preferred reporting items for systematic reviews and meta-analysis protocol statement guidelines.^[11,12]^

### 2.1 Literature search strategy

**Figure 1:**
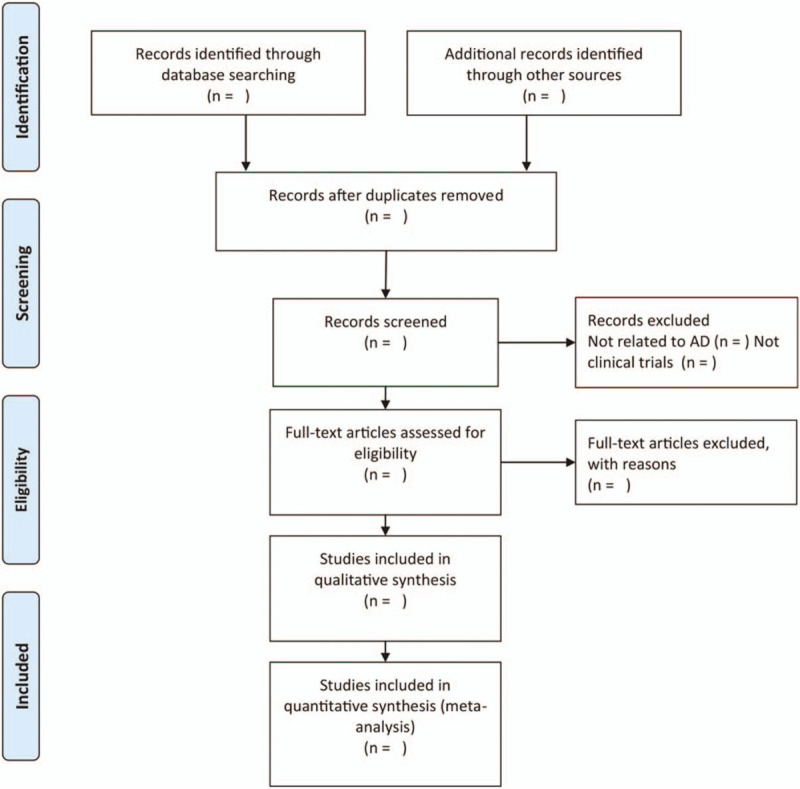
Flow diagram of literature retrieval.

An electronic search of 3 databases (PubMed, Embase, and the Cochrane Library) was conducted from their inception to May 2020 using the following keywords

(“mantle cell lymphoma”) and (“bortezomib”) and (“chemotherapy”) and (“randomized controlled trial”). In addition, the references of relevant articles were hand-searched for records that may have been missed. The study selection procedure is presented in a preferred reporting items for systematic reviews and meta-analysis flow chart (Fig. 1).

### 2.2 Criteria for inclusion and exclusion

The eligible studies need to conform to the following inclusion criteria:

(1) Randomized control trials (RCTs) enrolled MCL patients who were newly diagnosed, previously untreated, in first complete remission, or relapsed;
(2) the trials provided sufficient data on bortezomib-based chemotherapy for MCL patients, including the hazard ratio of the overall survival and the PFS or the odds ratio of the clinical-pathological factors, which could be calculated along with the corresponding 95% confidence interval;
(3) if data were presented in more than 1 article, the most recent or the most elaborate study would be selected;
(4) reviews, case reports, editorial comments, or letters to the editor without original data were not included.

### 2.3 Data extraction and quality assessment

Two investigators will independently extract data on the characteristics of the included studies (eg, first author name, publication year, intervention types, sample size), and they will assess the risk of bias (ROB) in individual studies by using the Cochrane Collaboration's Tool in the following aspects: The assessment includes sequence generation; allocation concealment; blinding of participants, personnel, and outcome assessors; incomplete outcome data; selective outcome reporting; and other sources of bias.^[13]^ Any differences between the authors on the data extraction and quality assessment will be resolved by discussion.

### 2.4 Statistical analysis

RevMan version 5.3 will be used to perform all calculations related to the meta-analysis. Dichotomous data will be calculated in terms of a fixed or random effect model and expressed by the risk ratio or odds ratio with 95% confidence interval. Time-to-event results will be expressed by hazard ratio and 95% confidence interval and pooled with an inverse variance method through a fixed effect model. Because overall response rate is not a minor probability event, it is usually expressed as relative risk. Adverse events will be generally expressed as odd ratio. The inconsistency index (*I*^2^) and the *χ*^2^-based Cochran Q statistic will be applied for heterogeneity detection between clinical trials. When assessing the difference in outcome, heterogeneity involving all trials will be examined. A value of *P* < .05 will be considered statistically significant.

### 2.5 Subgroup analysis

When there is obvious heterogeneity among included studies, we will perform a subgroup analysis in accordance with different study qualities, treatments, controls, and outcome measurements.

### 2.6 Sensitivity analysis

In the case of sufficient trials data, the ROB tool will be used to assess methodological quality. If low-quality articles are deleted, a second meta-analysis will be performed. The results and effect size of the 2 meta-analyses will be compared and discussed.^[14]^

### 2.7 Reporting bias

When there are at least 10 included RCTs, we will conduct Funnel plot and Egger regression test to identify any possible reporting bias.^[15]^

### 2.8 Grading the quality of evidence

In this systematic review, the quality of evidence for the entire study is assessed using the “Grades of Recommendations Assessment, Development, and Evaluation” standard established by the World Health Organization and international organizations.^[16]^ To achieve transparency and simplification, the Grades of Recommendations Assessment, Development, and Evaluation system divides the quality of evidence into 4 levels: high, medium, low, and very low.

### 2.9 Ethics and dissemination

No individual patient data will be involved in this study; thus, no ethic approval is needed. We will publish this study at a peer-reviewed journal.

## 3 Discussion

A numerous RCTs have reported that bortezomib in combination with chemotherapy for patients with MCL. However, their results are still not consistent. Therefore, the purpose of this study is to update and to determine the effectiveness and safety of bortezomib in combination with chemotherapy for patients with MCL.

There are strengths in our study. First, this meta-analysis provides a comprehensive assessment to whether bortezomib in combination with chemotherapy is beneficial for mantle cell lymphoma. Further, RCTs will be included in our studies and appear to be high quality and low ROB. However, there may be some limitations in our meta-analysis. This study may still have 2 limitations. First, some trials may have small sample size, which may affect results of this study. Second, the overall quality of some studies may be still low, which may impact study findings.

In conclusion, this study will help to determine the beneficial effects and harms on bortezomib in combination with chemotherapy for mantle cell lymphoma.

## Author contributions

**Conceptualization:** Xiaoxia Wang, Wen Li, Jing Li.

**Data curation:** Xiaoxia Wang, Wen Li, Xin Wang, Xiaoli Liu.

**Formal analysis:** Xiaoxia Wang, Wen Li.

**Funding acquisition:** Jing Li.

**Methodology:** Xiaoxia Wang, Wen Li, Xiaoli Liu.

**Project administration:** Xiaoxia Wang, Cuijuan Feng, Yihan Li.

**Resources:** Cuijuan Feng, Yihan Li.

**Software:** Xiaoxia Wang, Wen Li

**Supervision:** Jing Li.

**Writing – original draft:** Xiaoxia Wang, Wen Li, Jing Li.

**Writing – review & editing:** Xiaoxia Wang, Wen Li, Jing Li.
